# Leveraging phone-based mobile technology to improve data quality at health facilities in rural Malawi: a best practice project

**DOI:** 10.1186/s12936-021-03742-x

**Published:** 2021-04-27

**Authors:** Tinashe A. Tizifa, William Nkhono, Spencer Mtengula, Michele van Vugt, Zachary Munn, Alinune N. Kabaghe

**Affiliations:** 1 Division of Internal Medicine, Department of Infectious Diseases, Center for Tropical Medicine and Travel Medicine, University Medical Center, University of Amsterdam, Amsterdam, The Netherlands; 2 Training and Research Unit of Excellence (TRUE), School of Public Health and Family Medicine, College of Medicine, University of Malawi, Blantyre, Malawi; 3 JBI, Faculty of Health and Medical Sciences, University of Adelaide, Adelaide, Australia

**Keywords:** Malaria, Electronic data capture, Information systems, Evidence-based implementation, Baseline audit, GRiP matrix, Endline audit

## Abstract

**Background:**

To further reduce malaria burden, identification of areas with highest burden for targeted interventions needs to occur. Routine health information has the potential to indicate where and when clinical malaria occurs the most. Developing countries mostly use paper-based data systems however they are error-prone as they require manual aggregation, tallying and transferring of data. Piloting was done using electronic data capture (EDC) with a cheap and user friendly software in rural Malawian primary healthcare setting to improve the quality of health records.

**Methods:**

Audit and feedback tools from the Joanna Briggs Institute (Practical Application of Clinical Evidence System and Getting Research into Practice) were used in four primary healthcare facilities. Using this approach, the best available evidence for a malaria information system (MIS) was identified. Baseline audit of the existing MIS was conducted in the facilities based on available best practice for MIS; this included ensuring data consistency and completeness in MIS by sampling 25 random records of malaria positive cases. Implementation of an adapted evidence-based EDC system using tablets on an OpenDataKit platform was done. An end line audit following implementation was then conducted. Users had interviews on experiences and challenges concerning EDC at the beginning and end of the survey.

**Results:**

The existing MIS was paper-based, occupied huge storage space, had some data losses due to torn out papers and were illegible in some facilities. The existing MIS did not have documentation of necessary parameters, such as malaria deaths and treatment within 14 days. Training manuals and modules were absent. One health centre solely had data completeness and consistency at 100% of the malaria-positive sampled records. Data completeness and consistency rose to 100% with readily available records containing information on recent malaria treatment. Interview findings at the end of the survey showed that EDC was acceptable among users and they agreed that the tablets and the OpenDataKit were easy to use, improved productivity and quality of care.

**Conclusions:**

Improvement of data quality and use in the Malawian rural facilities was achieved through the introduction of EDC using OpenDataKit. Health workers in the facilities showed satisfaction with the use of EDC.

**Supplementary Information:**

The online version contains supplementary material available at 10.1186/s12936-021-03742-x.

## Background

Malaria was responsible for 229 million cases and 409,000 deaths in 2019 with more than 90% of both cases and deaths occurring in Africa [1]. In both low and high transmission settings, there are geographical and seasonal variations of malaria burden [2]. Individual, household, community and environmental factors affect the distribution of malaria infection. Identifying and addressing these variations may inform targeted interventions to reduce the burden of malaria in affected communities.

The World Health Organization’s (WHO) Global Technical Strategy for Malaria 2016-30 (GTS) suggests the transformation of malaria surveillance into a core intervention which would allow the capture of essential malaria data on a continuous basis thereby improving response to outbreaks, assessment of disease trends and optimization of responses [3]. This strategy further explains that surveillance of malaria should be utilized to inform appropriate action [3]. Strong surveillance systems linked to effective responses are critical for malaria elimination [4, 5]. The process of data collection together with reporting, active case finding, and linkage to response must be swift in endemic settings for proper identification of infections, prevention of ongoing transmission, and decrease transmission efficiency of vectors [4, 5]. In a majority of sub-Saharan African (SSA) countries, routine malaria surveillance involves collecting and reporting aggregate data from public health facilities through the national health management information systems (HMIS) [6]. Generally, malaria burden is monitored using household surveys or routinely collected health information. Household surveys are costly to conduct and analyse, and are usually based on a cross sectional design in a proportion of the population [7]. The cross-sectional design does not allow monitoring of burden over time.

Data collected in health facility registers for patients tested for malaria are valuable for monitoring trends in malaria morbidity and evaluating the impact of malaria control interventions [8]. However, the data may be incomplete, unreliable and paper-based and, therefore, prone to errors during manual tallying [6]. In addition, facilities may use several registers for a single patient making it complex to easily access individual patient information [9]. When it comes to reporting from primary health-care facilities to secondary health-care facilities, these data are aggregated by test result and age category; the aggregated reports lose valuable information including its location.

Most health facilities in Malawi use paper-based registers to record and report routine malaria information. The paper-based registers are also used to record other illnesses at the facility. For each illness, the records are tallied and compiled into an aggregated report, which is physically sent to the district health office. The reports from all health facilities in the district are entered into the district health information system (DHIS) using a computer. This system, as already elaborated above presents a number of challenges more especially with errors that have not been addressed during primary data collection.

Malawi’s national malaria operational plan includes goals to improve the country’s capacity to collect and use information and to adapt to changing epidemiology and incorporate new tools by 2020 [10]. For monitoring progress towards achievement of these goals, a system for capturing both local and regional transmission is essential. Particularly, there is a need for an effective surveillance system that can monitor near real-time malaria data, target focal areas of infection, increase capacity to identify transmission hotspots and rapidly identify changes in malaria transmission, morbidity and mortality.

In this paper, a description of an evidence-based quality improvement project for a malaria information system conducted at four health facilities in a rural community of Chikwawa in Southern Malawi was done. The overall goal was to develop and deploy an evidence-based best practice system to improve the quality and use of the health information system. Lessons learned and experiences related to implementation of the system were discussed.

## Methods

### Study design

The JBI approach to evidence implementation was followed [11], including the use of the Practical Application of Clinical Evidence System (PACES) software [11] and Getting Research into Practice (GRiP) framework for promoting evidence based health care following attendance at an evidence implementation training programme, funded through a competitive scholarship [12–14]. This approach involves three phases of activity:

1. Establishing a team for the project and undertaking a baseline audit based on criteria informed by the evidence.
2. Reflecting on the results of the baseline audit, providing feedback and designing and implementing strategies to address non-compliance found in the baseline audit informed by the JBI GRiP framework.
3. Conducting a follow up audit to assess the outcomes of the interventions implemented to improve practice, and identify future practice issues to be addressed in subsequent audits.

### Project site

The project was conducted from September 2018 to January 2019 in 4 health facilities namely Chapananga, Kakoma, Kapichira and Majete 1 in the southern Malawi district of Chikwawa. The facilities are situated within the catchment area of the Majete wildlife reserve (MWR). This project site has been previously described [15], but briefly the catchment area is characterized by highly heterogeneous land use for subsistence farming and small scale business. Chikwawa has a hot and humid tropical climate for most of the year. Some of the services offered in these facilities include out-patient department (OPD), maternity, under-five clinic and immunization.

### Ethical considerations

The project was registered as a quality improvement activity within the health centres, and therefore did not require ethical approval. Approval to conduct the project in Chikwawa district was however provided by the Chikwawa District Health Office.

### Data collection

The WHO established standard protocols for monitoring and surveillance of malaria treatment, both in children and adults, specifically from low and middle-income countries (LMIC) [16, 17] helped in coming up with this approach. The literature was explored to obtain evidence on the best available practice for electronic monitoring of the quality of malaria treatment in children living in LMIC which was synthesized in an evidence summary and informed the criteria development. The data collection process was categorized into four phases, with these phases progressing in sequential order.

### Phase 1: team establishment and baseline audit

The first phase of the project was to form a core project team to provide technical, logistical and strategic guidance. During this phase, a team to coordinate all project activities was established and comprised of health care workers (HCW) from Chikwawa district health office (DHO), particularly data clerks and medical assistants. Data clerks are a cadre within the Malawian health system whose role is to conduct data entry and aggregation at specific health facilities. On the other hand, medical assistants are a cadre of clinicians within the health system, whose role is to provide primary care of patients in primary health facilities (health centres and dispensaries). All the four implementing health centres had these cadres. Some members from the DHO that were incorporated include the District Medical Officer (DMO) and the health management information system (HMIS) officer. Others team members included information technology specialists, masters’ student, PhD student and a postdoctoral researcher all working with the College of Medicine, University of Malawi. DHO personnel provided support in engagement of the health centres. Health centre personnel were to be the implementers of the project. The composition and roles of personnel are summarized in Additional file 1: Appendix 1.

After forming the team, audit criteria were developed (Table 1). A structured questionnaire was created and administered to the health facility in-charges (usually medical assistants) and data clerks to assess the existing health facility information system. Furthermore, documentation of roles and responsibilities of personnel at each level of the data monitoring system was checked. Questions concerning the existence of training manuals and modules for the monitors together with the type of infrastructure at each facility were raised.

**Table 1:** Shows the evidence informed audit criteria used in the project (baseline and follow-up audit) together with a description of the sample and approach to measuring compliance with best practice for each audit criterion

Audit criterion	Sample	Method to measure
eMIS is used for quick access to both individual and aggregated data	4 Health facilities	Interview health facility manager
MIS includes suspected, tested and confirmed cases	4 Health facilities	Checklist for OPD, laboratory and Artemisinin-Lumefantrine (AL) registers
Monitoring system includes location of information	4 Health facilities	Checklist of OPD register
Monitoring system includes prior treatment (within 14 days)	4 Health facilities	Checklist of OPD register
Monitoring system includes malaria treatment and dose information	4 Health facilities	Checklist for OPD & anti-malarial registers
Data in monitoring system is complete	25 Malaria case records at each facility	Check retrospective records in OPD, laboratory and anti-malarial registers
Internally consistent with no duplications in monitoring system	25 Malaria case records at each facility	Check retrospective records in OPD, laboratory and anti-malarial registers
Roles, responsibilities, training manuals and modules available	4 Health facilities	Interview health facility manager

In order to determine prior treatment of malaria within 14 days, a checklist was also developed for OPD, laboratory and anti-malarial registers. In addition, records of 25 randomly selected malaria positive patients in each facility were checked for completeness and internal consistency. Completeness was assessed by checking that all required data elements had a recording while consistency was assessed by comparing the data in all the three registers. This audit required a total of two days with a maximum of three hours at each site for it to be completed.

### Phase 2: design and implementation of strategies to improve practice: GRiP strategy

After obtaining the baseline audit results, the project team identified strategies that would help address the bottlenecks in data management. The team developed an electronic case report form (eCRF) based on the existing malaria, rapid diagnostic tests and OPD registers ensuring that the eCRF (Additional file 1: Appendix 2) was a digital replica of the paper based forms (Additional file 1: Appendix 3). The eCRF was designed using (Extensible Markup Language) XML script, accounting for all the variables which were on the paper-based register and available evidence for electronic monitoring of the quality of malaria treatment in children in low and middle-income countries. Testing of the eCRF was first done by the project team, to ensure that all skip logic functioned, and all the required data and variables were being collected by the electronic form. In order for data collection to occur at the health centres, Samsung android tablets from Majete malaria project were installed an application called Open Data Kit (ODK); the eCRF was uploaded and configured to run as a client for data collection on these Samsung tablets (Additional file 1: Appendix 4). It was an opportunity of having a research project that had been using Samsung android tablets for data collection. Permission for use of these devices in the facilities was granted by the project. Electronic data capturing (EDC) was to take place in the health facilities with Samsung android tablets having ODK software. ODK is an open source android application, which can be used to capture disaggregated individual records using mobile devices; the records can be sent remotely to a server or desktop and is immediately accessible using a specific Uniform Resource Locator (URL) and appropriate log in credentials. ODK can collect text, numeric data, images and geographical coordinates in resource limited settings by people with minimal education qualifications [18]. ODK can run both on Windows machines and Android tablets, however, android tablets were selected because they have superior battery life and require minimal technical knowledge to operate as compared to windows machines.

In addition to Android tablets and Windows, ODK can also run on MacOs and iOS using Kobo toolbox, Enketo and GIC collect [19]. These applications support offline data collection and form submission [20]. Considering that all the ODK collect alternatives are used in survey-based data gathering, works without network connectivity and manages a very large range of question and answer types like ODK collect, it was possible to use the iOS for data collection, however the latter is too expensive for this environment and it would require a lot of technical investment.

Electronic data capturing systems have been used across Africa, in various forms. A systematic review looking at the use of EDC in African settings revealed that 91% reported use of Open Source healthcare software, with OpenMRS being the most widely used [21]. Experiences of using EDCs tend to be similar across the continent with major successes being greater data accuracy, improved timeliness, availability of routine reports and reduced data duplication [21–23]. Despite these successes, challenges have also been documented on the use of EDCs in sub-Saharan Africa; these include high cost of set-up and maintenance of EDC, low level of information and communications technology (ICT) literacy, poor infrastructure and power outages [21, 23]. EDCs have been piloted and used in Malawi before mostly in research settings. In a study by King et al., four different EDC systems were piloted in rural areas with the aim of highlighting advantages and disadvantages of each system through personal experiences of fieldworkers, project managers, and EDC system developers [24]. They have also been used at tertiary health facilities (central hospitals) mostly in the form of electronic medical records (EMR), however they usually look at one condition [25, 26]. The most common one is the EMR in the form of a touchscreen system at the point of care (POC) to monitor and support antiretroviral therapy (ART) scale up [26]. This was first piloted in April 2006, at Queen Elizabeth Central Hospital (QECH), a specialized and referral hospital, located in Blantyre City, southern Malawi, and scaled-up to other tertiary institutions in the subsequent years [26]. With the efficiency of EDC in data collection and transmission in research studies, that the authors have had an experience in, and the few scenarios of use at tertiary level of care, it was necessary to incorporate the suggestions raised by the stakeholders from the health facilities and the district management team during the meeting to use a form of electronic data capturing. This would try to provide a solution to the problems of data management.

A meeting was scheduled with health care workers (medical assistants and data clerks), HMIS officer and DMO, to discuss the gaps and challenges identified with the existing system through the baseline audit, and the proposed evidence-based practice for malaria information system. There was an orientation and feedback session based on the proposed system. During the discussion and orientation, possible challenges were explored to the use of EDC and the strategies and resources required to address them. A large concern was regarding the battery lifespan of the devices and the additional workload required to concurrently implement both paper and electronic systems.

Specific attention was given to the facility data clerks, since they are the ones that would be using the tablets to enter data. It was ensured that they understood how to conduct the data collection, and once completed, be able to send all the collected outpatient data (not only malaria patients) for their respective facilities to the central repository housed at the district health office of which the HMIS officer had administrative rights. The existing system of data entry and aggregation where patients’ information and treatment is entered into the registers after they have been assisted at the point of care (POC) and all the necessary procedures and treatment have been done was adopted. Data clerks used the tablets for data entry at these aggregation points. During the meeting, emphasis was placed on the need to follow all the necessary procedures and documentation for the management of patients at health facilities, despite the introduction of the EDC. When it comes to documentation at the health facilities, patient’s health booklets are the essential data tools and they form an interphase between the various service points in the facility. In the study sites, when a patient was seen at the POC, the medical assistant documented the necessary plan concerning the patient in the patient’s health booklet. This was then taken by the patient to the next POC, e.g. the laboratory where the plan documented by the clinician such as malaria testing was executed by the laboratory staff. Afterwards, documentation of the results in the patient’s health booklets was done by the POC. The patient would then follow-up with the clinician and the clinician would write the necessary plan or treatment depending on the results to either be received at the pharmacy within the facility or purchase, depending on the circumstances, in client’s health booklet. After receiving the treatment, the patient would present the health booklet to the data clerks where information was entered into the eMIS and the registers.

Specific users such as the data clerks, DMO, were given access to the server and had some full control rights. The project team were assigned read only privileges to view the data only and monitor the data as it comes in. In addition, data cleaning scripts and report generating scripts were written to assist and automate the monthly report for each facility, as this was previously manually tallied by the data clerks on papers and transported via vehicle to the DHO office for data entry in their system.

The steps followed when coming up with the GRiP strategy were essential as they would help highlight the steps required in order to have a successful implementation programme that is acceptable by everyone. The whole team specifically the users proposed the adoption of a mobile based EDC using the ODK application.

### Phase 3: follow up audit post implementation of change strategy

After implementation of the data collection tools, a follow-up audit of the health facilities was made. Data entered into the local server was checked for various parameters as assessed in the baseline audit.

### Phase 4: end of survey assessment

A questionnaire for EDC assessment was developed which was used at the end of the survey to document the experience of users of the system.

## Results

It was discovered that the current data capture system involves the use of registers in all facilities. There are a number of registers the Malawi government through the Ministry of Health developed and allocated to all the health facilities. Some of them include the out-patient (OPD) department, anti-malarial, laboratory, under-five and maternity registers. Since the main condition of interest was malaria, assessment of the OPD, laboratory and anti-malarial registers was done. During phase 1 when assessment of the health facilities was conducted, it was discovered that the current paper-based system had problems which included:

1. Torn and lost papers within the registers.
2. Illegible print (Additional file 1: Appendix 5).
3. Poor documentation by providers.
4. Use of multiple registers to capture data of an individual.
5. Filled up registers occupied a lot of space in the facilities reducing operational capacity (Additional file 1: Appendix 5).

Table 2 below summarizes comparisons between baseline and follow-up audits. It shows that there was no existence of electronic management information system (eMIS) at the beginning of the survey compared to the end. The malaria information system (MIS) used at all health centres did not have some necessary parameters, which include captured deaths from malaria and malaria treatment within 14 days.

**Table 2:** Baseline and follow-up audit outcomes

Health Centre	Majete		Kakoma		Kapichira		Chapananga	
	Base	End	Base	End	Base	End	Base	End
eMIS is used for quick access both individual and aggregated data	No	Yes	No	Yes	No	Yes	No	Yes
Includes suspected malaria, tested and confirmed cases	Yes	Yes	Yes	Yes	Yes	Yes	Yes	Yes
MIS includes deaths from malaria	No	No	No	No	No	No	No	No
MIS includes patient location	Yes	Yes	Yes	Yes	Yes	Yes	Yes	Yes
MIS includes treatment (within 14 days)	No	Yes	No	Yes	No	Yes	No	Yes
MIS includes malaria treatment and dose	Yes	Yes	Yes	Yes	Yes	Yes	Yes	Yes
Roles, responsibilities, training manuals and modules available	No	No	No	No	No	No	No	No

### Baseline and follow-up audits

**Fig. 1:**
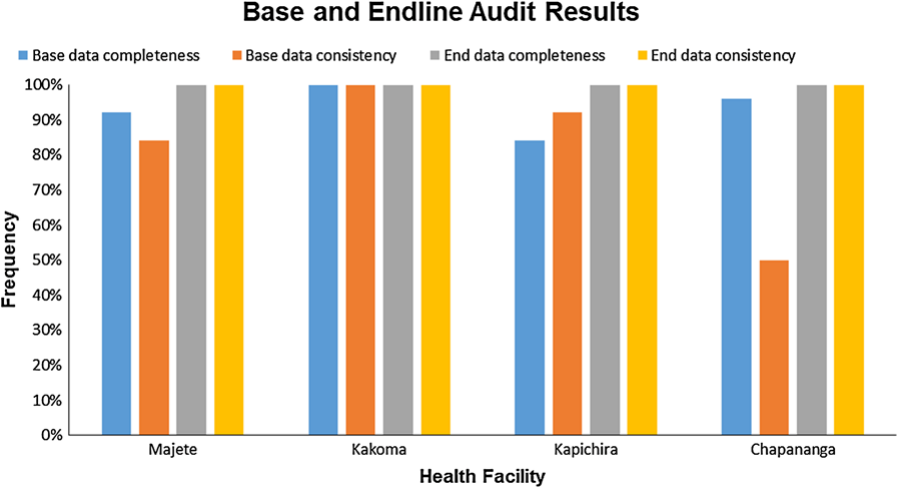
Compliance with best practice audit criteria in follow up audit compared to baseline audit (%)

Figure 1 shows results for data completeness and consistency at baseline and follow up audits for 25 randomly selected records in the four health facilities. During the baseline audit, Kakoma was the only health centre that had data completeness and consistency at 100%. Majete 1 and Kapichira had data consistency and completeness both above 80%. Chapananga had the least percentage in data consistency, 50% at baseline but attained 100% after the audit.

### Getting research into practice (GRiP) strategy for identification of the barriers, strategies in addressing the barriers, and resources used to reach the intended outcome

The first barrier identified as impeding the success of EDC implementation was a lack of support from the district health office (DHO). During the baseline audit HCWs at health facilities expressed concerns that the DHO's response to some issues or problems arising from some health facilities is generally slow. To find a solution to this problem, it was agreed that all parties involved should meet and discuss possible solutions. There had to be a mutual benefit for both parties, and the most likely one was that, because this would be an innovation, Chikwawa would be the country's pioneering district for this innovation. The resources required for this meeting were the transportation of all participants from the health facilities to come for the EDC demonstration. At the end of the meeting, all issues were resolved, and the piloting of EDC implementation in selected health facilities in the district was approved.

Second, it was discovered that health facility data clerks may be hesitant to use EDC while maintaining registers because it would add to their workload. To overcome this situation, the proposed strategy was to engage the DHO and health facility managers during the baseline audit and orientation and explain the significance of this pilot. Orientation meetings in which health facility personnel were trained on EDC and provided solutions to problems were held. The goal was met when electronic data was successfully collected and safely transmitted to the server.

Third, it was discovered that there was a lack of technical capacity among health facility data clerks to implement EDC. It was proposed that EDC be given orientation and training so that data clerks can be involved in the design of data collection forms for ease of use. Furthermore, technical assistance would be provided as needed. The project team provided electronic tablets to ensure the success of the proposed strategy. In addition, the project team and data clerks created simplified electronic forms. Training on how to use the tablets was provided during the orientation. Supportive supervision was provided by the project team during the data collection process. As a result of this, electronic data was successfully collected and safely transmitted to the server.

### Follow-up interview findings

Users from all the facilities indicated that the EDC system was easy to use together with the use of electronic tablets and it made their work easier. They further established that the EDC improved their productivity and quality of care compared to the paper-based system. There were different views among users on whether the solutions that were developed and implemented met the intent of the promise. Some agreed with others highlighting that there were a number of changes made on the ODK form. Advantages that were mentioned by the users concerning EDC include:

1. Easy accessibility of data.
2. Fast.
3. Easy storage and management of data.
4. Easy to transmit and send data.

Despite this, the following disadvantages were mentioned:

1. The system required power supply, this posing a challenge at sites which had no access to the national grid.
2. Errors were noted in the ODK ranging from wrong spellings of villages/medication, improper dosages of some medications. These occurred when creating the ODK form.
3. Possible theft of the devices.
4. Difficult to send electronic data to district health office because of lack of phone airtime needed for a wireless connection.
5. Could not access the data to compile reports.

### Local backup and data storage

The need to backup data at each health facility is essential. From the baseline audit results, it was identified that Chapangana, Kakoma and Majete health facilities did not have a laptop or computer facility. With these findings, it was difficult to have a backup of the tablet every day. This raised an on-going problem when the data was not sent to the server the same day, the date would be stored in that tablet until the data was sent to the central server. Solutions to mitigate these problems were suggested by the team. The initial plan was to assign airtime for internet to the data clerks, however there was an oversight during budgeting, therefore, making it not feasible to allocate airtime to individual data clerks. To resolve this, a discussion was done with the HMIS officer at the DHO so that data collected on a weekly basis in the tablets should be sent to his office by assigning the data officer, to travel to the sites and send any data which was collected that particular week. All sites were visited at least once within the week. The need to mount servers in these hard to reach areas that lack of essential utilities such as electricity at the health facilities was just not feasible.

As far as data backup at the central system is concerned, the central system was set up to conduct daily backups scheduled to run at 3:00 pm during the day. A full backup of the database was automated to copy the database files to a network attached storage (NAS) device. Every morning, the master’s student checked that the previous backup was successful by verifying that a backup was performed comparing the file sizes of the current backup with any previous backups.

In an event that a backup failed, a manual backup was performed immediately and the backup issue was reported for troubleshooting. Additional manual backups on the central system were done on a weekly basis by securely connecting to the server and then copying the Structured Query Language (.sql) dump to an external hard drive for safe keeping away from the main storage site. The last Friday of the month, a manual backup would be performed by the master’s student near the end of the day to ensure that there was a copy of the database in its raw state for every month.

## Discussion

This quality improvement project in rural Malawi is the first to utilize mobile devices on ODK platform to capture patient-level data for disease surveillance at sub-district level. The conditions of the location selected for this implementation project are comparable to other malaria endemic countries, and are characterized by being LMIC, dependent on subsistence farming, with reduced access to road networks, infrastructure, running water, electricity.

Information systems, particularly at point of care, need to be simple and sustainable and not overburden health delivery staff. This study demonstrated that HCWs were able to reliably record and transmit data through an electronic reporting platform in this scenario, EDC using ODK on tablet software. With the use of EDC, capturing and incorporating of some essential parameters that had not been developed and incorporated in the paper-based registers were done. Completeness and consistency of data in EDC was markedly better compared to the poor levels observed using the paper-based system. This could be attributed to the fact that manual entry of the paper-based registers, may have resulted in some reports being lost in the process, mix-up of entries as data clerks are responsible for handling multiple registers or no entry at all into the database due to other problems such as lack of stationery. Similarly, use of multiple registers to capture a single entry could have contributed to mix-up of entries as a wrong result could be given to a patient. Having many forms and registers being handled by a single HCW may contribute to having fatigue and loss of focus thereby resulting in having inaccurate data and illegible writings as seen in most of the health facilities. Furthermore, this can complicate the situation as it cannot be clearly established whether what has been reported reciprocates to what the HCW does.

This EDC method offers a better solution to the use of multiple registers by a single individual. Before data collection when forms were being developed, sampling of multiple registers used in entering malaria data, such as the OPD, laboratory and anti-malarial registers was conducted. These registers were adapted with a few additions and developed into the electronic format that was placed in the devices for data clerks to use. This resolved the problem of having data entry in multiple registers by a single individual thereby avoiding unnecessary errors. Similarly, this could also be adapted to incorporate other registers that are used at the health facilities such as the antenatal and immunization registers.

It could be argued that improving the current paper-based systems could be more affordable and bring in better results. Successes on the improvement of existing paper-based systems for data collection have occurred in different areas within sub-Saharan Africa [27–30], and innovations included having simple registers and combined primary care registers. Despite this, there are still existing challenges that are still being addressed in those setups, which within the current setup also apply. These include existence of inadequate human resource, space for storage and frequent trainings for HCW responsible for data management. Thus, paper-based form would currently be a challenge as a number of impending issues need to be addressed such as having adequate human resource, additional storage facilities, and transport issues where aggregated reports need to be sent to the district office for tallying and uploading into the HMIS. It would be easy to scale up the improved paper-based system as it will be just a replacement of the old registers with new ones.

Use of the EDC system in the current deployment eliminated the need for paper records by having all the required indicators pre-programmed into electronic forms and inserted into the ODK using Samsung tablet devices. It was easy for all HCWs to ensure that standard protocols are followed and the system itself was designed in a way as to not miss any field. This design facilitated complete records and proper storage. This system further provided an opportunity for remote quality control, as the data once collected could then be transferred from the remote server where other checks could be done to ensure consistency and completeness. The need to have EDC would also assist in reducing the problem of storage of records. In general, space that would be useful for service provision in rural health facilities could be turned into storage for filled up registers, so with EDC this problem could be overcome.

In this setting, that is malaria endemic, it has been shown that case registers were available but the data was mostly unreliable as it was incomplete, error-prone and experienced delays in transmission to the district site where all data for the district were compiled and aggregated. This makes it possible for such registers to under-represent the burden of disease within the community. Quality and timely data at district level is essential as it would help to identify areas with the highest burden so that proper intervention coverage, such as the use of ITNs or mass drug administration, can be implemented. It also helps to identify hotspots that are areas of high endemicity and are capable of causing an epidemic if not properly managed. Quality and timely data help to prioritize the allocation of resources in areas that are heavily burdened by the district in order to obtain adequate supplies of medicines, such as anti-malarials, good public health practice in order to identify cases in the most remote areas in order to avoid mortality.

In the implementation effort reported here, EDC showed many attractive features and was highly regarded by HCWs in the field as a useful tool to increase their efficiency, and by supervisors as a great resource for quality control. Despite the achievements brought through the introduction of EDC, challenges of this system were also observed. Firstly, this system is effective mostly at sites where there is a reliable source of power to charge the tablets. Some facilities however did not have electricity as a result managing the devices was somehow difficult and the team had to find other means to avoid them shutting down. This situation is more complex in hard to reach areas where personnel need to travel long distances to get to a point where they can have access to power and it is typical for most rural areas in LMIC. For a programme of this nature, access to power is very important for successful implementation. Possibly, cheaper forms of power such as solar electricity could be the solution for rural areas in order to address such problems. These solar panels have been successfully used elsewhere [31].

Another challenge raised was that devices are mostly prone to theft. There is a greater need to safeguard these devices otherwise implementation of the programme would not be a success once these devices have gone missing. Typically, there is need to find ways of securing them mostly when they are not in use with means of tracing them. Since it was a pilot, one of the concerns that was also raised was the addition of workload as a result of adding EDC together with the paper based system. HCWs had to switch between use of registers and EDC so that data was ably captured using both data capture methods. There is also the problem of high turnover at most government facilities so a concern was raised that there would be need for orientation for new staff who would be responsible for data entry. For a successful project and sustainability of the implementation programme, there is need for continuous engagement and orientation of all stakeholders, which at times can prove to be costly [32].

Facilities also mentioned that they experienced challenges with airtime to send data to the server, they would rely on finding other means of transport to the DHO so that data reaches the server. Through this discovery, it enabled us to discuss with the teams involved on better ways of mitigating these problems. There is need to find the best ways possible for the data to reach the server. This could require investing into the provision of adequate airtime so that data transmission should not be affected. As described by Proctor et al., adoption of an intervention best resides from the perspective of the provider [32], in this case, engaging the stakeholders was of great significance to the solutions of the problem. Facilities also were concerned that data being collected was not easy for them to access once sent to the DHO. This problem was due to the fact that access to the server was only limited to the administrator. It can be easily resolved by providing access to other service providers, such as the data clerks and medical assistants from the facilities to have access to the server’s URL.

In terms of the costs of implementing this EDC system, the start-up costs, which are mainly for software, form development, as well as the hardware need to be considered. In this case, since ODK collect is free and an open source software, the initial investment will be small. It is a software that is favoured in areas with poor internet connectivity, such as the pilot area, since the entire process, from form creation to data download, can be completed offline. Open source software, such as ODK, is affordable and allows for adaptation and local use. The need for data cleaning is considerably reduced because EDC has in-built cleaning rules. This saves time and costs by reducing the need for field verification, and manual data checking and correcting. When it comes to the hardware, the startup costs will be high because devices such as android tablets will be needed for various health facilities. The android tablets used in the pilot were under $100 (US Dollars), and with advances in technology and availability, prices are likely to continue to decline. While EDC has higher start-up costs than paper-based system in this case, there are no data entry, printing, or photocopying costs for EDC. However, EDC running costs required to sustain the system need to be taken into consideration. As time progresses with the use of EDC, issues of wear and tear begin to surface. Situations may arise such as the need for replacement of damaged, lost, or stolen hardware (including tablets, chargers and other supporting equipment). Institutions and facilities must assess the EDC system on a regular basis in order to determine whether damaged equipment should be repaired or replaced entirely. This is very important because it will help to avoid hiccups in the data collection process. These costs must be properly determined and taken into account prior to a massive scale up, as this will assist the government or facilities in preparing. Other operating costs include managing internet or cellular connectivity, as well as ICT costs for managing an ever-growing database and server capacity, which is a challenge in most LMIC. Cost aspects were however beyond the scope of this survey as it was formulated to come up with possible solutions of having better data quality in the four rural health facilities.

The outcome measurements of the project met the expectations to the stakeholders in terms of accuracy, completeness, timeliness and transmission for feedback and support of HCWs in a remote setting in Malawi. Implementation of a system such as EDC using ODK on a larger scale, such as a whole district level, is therefore warranted and needs to be implemented in Malawi. This could be a reliable indicator of the feasibility of the system in those that would be using it and can be a determining factor for the scalability of this innovation. Some recommendations that were made included that refresher trainings should happen and that health facility in-charges should be involved when developing the ODK form to minimize errors. There is also need for proper planning for the project together with the district management team on how best EDC can be executed at a large scale. Since it is not yet adopted for use by the government at its facilities, the need of extra human resource such as additional data clerks to minimize the workload incurred with the introduction of EDC needs to be addressed. For proper continuity there is need for a proper timeframe to be put in place for the large-scale pilot, such as at district level, results need to be discussed and disseminated with all the key stakeholders, including policy makers. There is also need for availability of devices that are working properly so as to avoid disruptions in data collection.

Scaling up an intervention is a meticulous process that needs buy-in from implementers, so it is usually gradual and needs input from various stakeholders including policymakers. Based on previous experience with the use of EDC in the form of EMR for ART in tertiary hospitals across Malawi, at least two pilots had been conducted to determine the extent to which EMR is best adapted by practitioners [26]. Shortfalls of the new system were noted and documented by implementers and an independent taskforce was set up to evaluate the implementation of EMR for ART [26]. It should be noted that there are contextual differences between using EMRs as an EDC method for capturing conditions like (HIV/AIDS) that require referral and specialized care at tertiary institutions, as was the case above, and using EDC with tablet software using the ODK method, as demonstrated in this pilot. Scalability to other sites would be slightly different than with the EMR system, as it would involve mostly facilities similar to the pilot facilities as well as different types of devices (tablets in this case compared to fixed touch screens as was the case with EMR) and equipment. Since this was a pilot that only assessed the quality of the data management systems within the four health centres, the intention of scaling up was not part of the agenda. However, with these findings, building on these results would be essential so as to see the outcomes of the scale up. The next steps would be to conduct a large-scale pilot at district level, involving all facilities including those that have better access to utilities, such as electricity, and are not in hard to reach areas.

Sustainability is a critical aspect in as far as a programme’s lifecycle is concerned. By definition, sustainability is defined as the extent to which a newly implemented programme or service is maintained or institutionalized within a service setting’s ongoing, stable operations [32]. In a situation where EDC with ODK is being implemented, just like any other programme, informing, educating and communicating (IEC) users about the objectives is very critical for creating and gaining buy-in to the user's vision of using it. There has to be consistent feedback between all stakeholders. At the end of the day, there has to be ownership of the whole system by the users since it is more effective compared to the previous system. In this setting, where EDC has not been used before it is necessary to implement this on a small scale first, e.g. a district-level trial for a specific period of time. Its successes and challenges need to be noted in order to get the users' perspective. If perceived as effective by the users, the workload must be eased otherwise early system glitches may compromise perceptions of reliability, ultimately undermining user buy-in.

## Conclusion

This work demonstrates that using mobile technologies, such as EDC using the ODK platform in the capture and reporting of health facility data in remote locations of LMIC provides timely, complete and high quality data for malaria and other common conditions. Quality, complete and timely data collection by health workers in a remote setting in Malawi is achievable and such data can be transmitted, aggregated and used by HCWs and the district to improve their service delivery. Investment in this system would benefit the health care system as witnessed from the results. The technology uses cheap software and with advances in technology the probability of having cheaper devices with time still exists. However, there is need to build up on the results through piloting on a large scale involving multiple sites at district level and to determine the cost effectiveness due to the infrastructure and other health system needs in similar settings. This will assist in providing further evidence on adaptability by users and possibly sustainability.

## Supplementary Information

**Additional file 1:** **Appendix 1**: Personnel and their roles in the project. **Appendix 2**: Electronic Case Report form (eCRF) adopted from registers. **Appendix 3**: Contents of the registers in its paper-based format which was later used and adopted for eCRF. **Appendix 4**: Samsung tablets with ODK software used. **Appendix 5**: Registers provided by the ministry of health, illegible print seen from registers, torn pages from registers.

## Data Availability

The dataset is accessible at the corresponding author upon a reasonable request.

## Abbreviations

AL: Artemisinin-lumefantrine
ART: Antiretroviral therapy
DHIS: District health information system
DMO: District medical officer
eCRF: Electronic case report form
EMR: Electronic medical record
GRiP: Getting research into practice
GTS: Global Technical Strategy for Malaria
HCW: Health care worker
HMIS: Health management information system
ICT: Information and Communications Technology
JBI: Johanne Briggs Institute
LMIC: Low and middle income countries
MIS: Malaria information system
MMP: Majete Malaria Project
MWRP: Majete Wildlife Reserve Perimeter
ODK: Open data kit
OPD: Outpatient department
PACES: Practical Application of Clinical Evidence System
POC: Point of care
SSA: Sub Saharan Africa
URL: Uniform resource locator
WHO: World Health Organization
XML: Extensible markup language
