# Supplementary material for: Leveraging phone-based mobile technology to improve data quality at health facilities in rural Malawi: a best practice project
Source: Malar J. 2021 Apr 27;20:203. doi: 10.1186/s12936-021-03742-x (PMC8077781; doi:10.1186/s12936-021-03742-x)

**ADDITIONAL FILES FOR LEVERAGING PHONE BASED MOBILE TECHNOLOGY TO IMPROVE DATA QUALITY AT HEALTH FACILITIES IN RURAL MALAWI: A BEST PRACTICE PROJECT**

**Appendix 1:** Personnel and their roles in the project

Table 1: Roles and responsibilities of personnel involved in the project

| **Name** | **Position** | **Role** |
| --- | --- | --- |
| Alinune Kabaghe | Team leader | Team leader/Coordination |
| Tinashe Tizifa | PhD student | Logistical support |
| William Nkhono | Data manager (postgrad) | Technical support |
| Spencer Mtengula | Data officer (undergrad) | Design + Training |
| Wamaka Msopole | District Medical Officer | Technical & implementation guidance |
| Ken Mkandawire | Health information system | Technical & implementation guidance |
| 4 health facility in charge | Medical assistants | Administrative support |
| 4 data clerks | Data clerks | Data entry |

**Appendix 2:** Electronic Case Report form (eCRF) adopted from registers


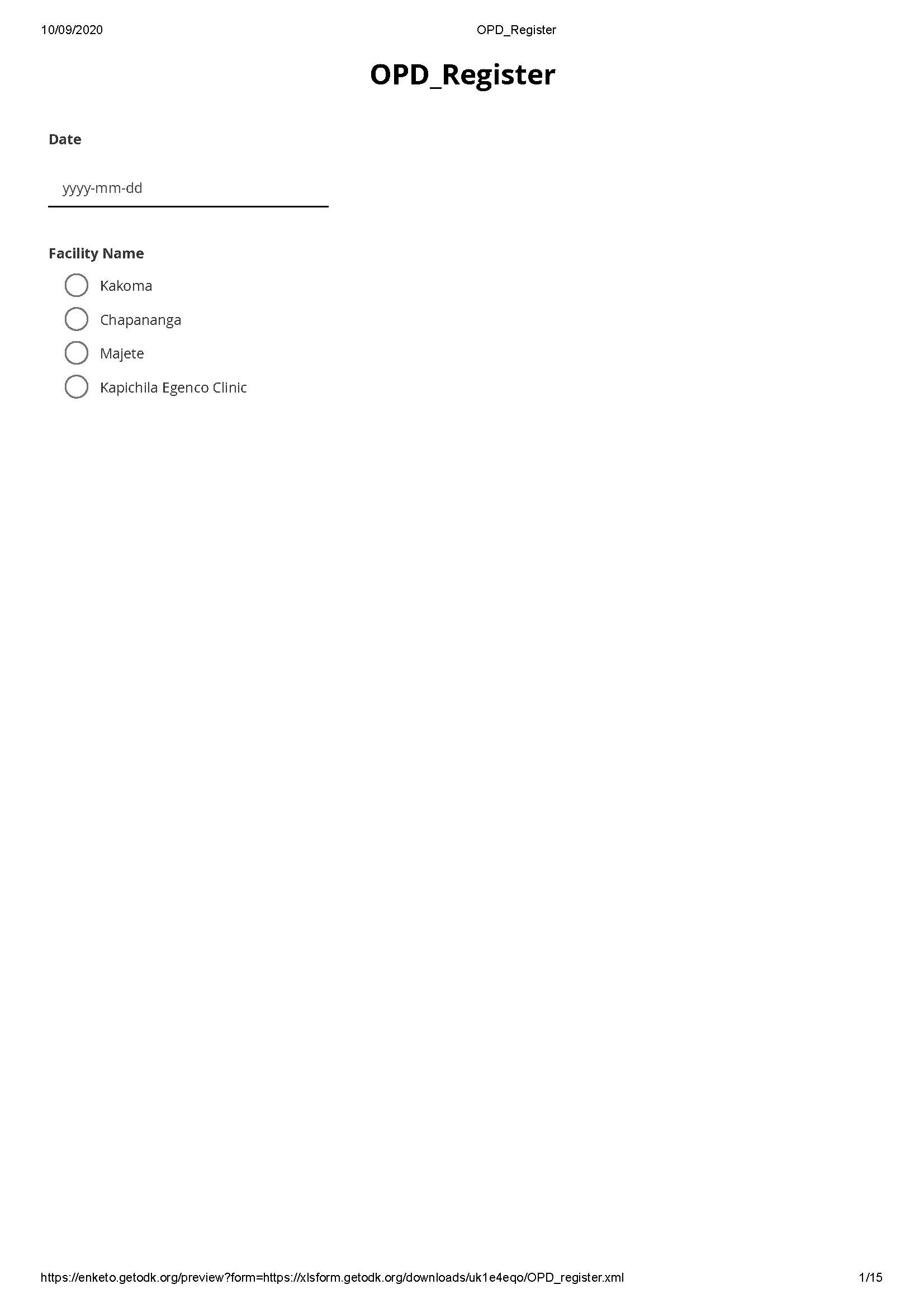


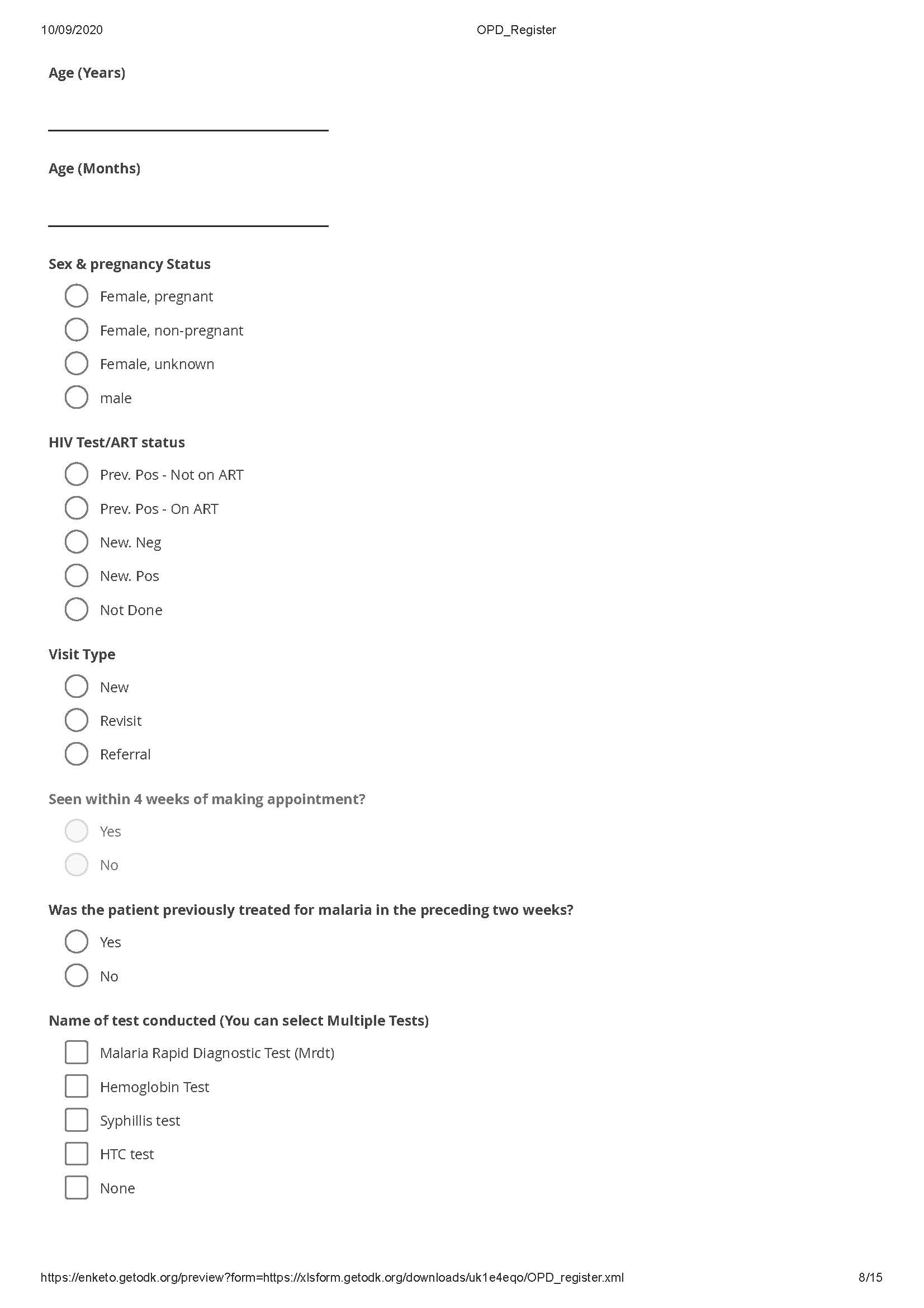


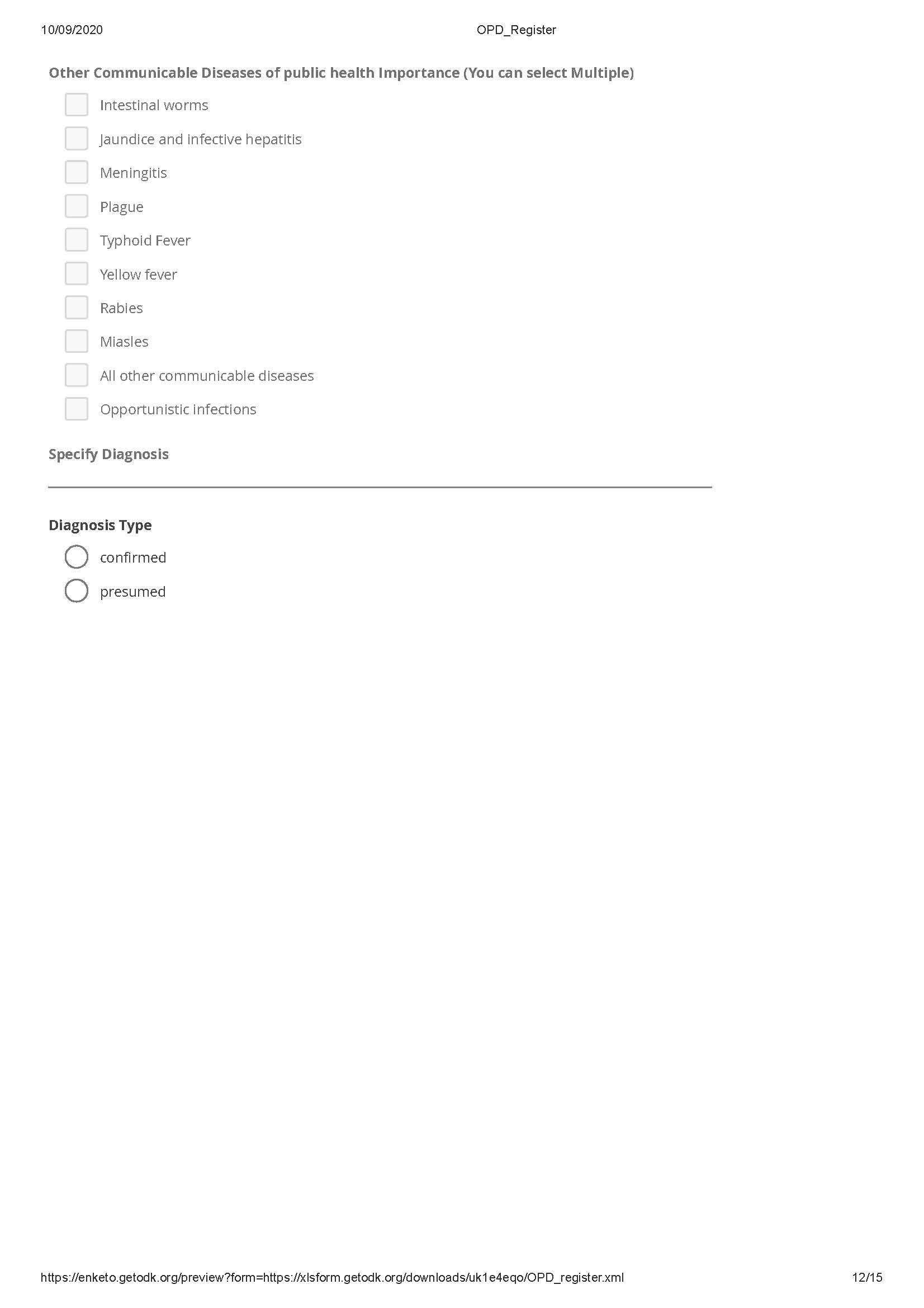

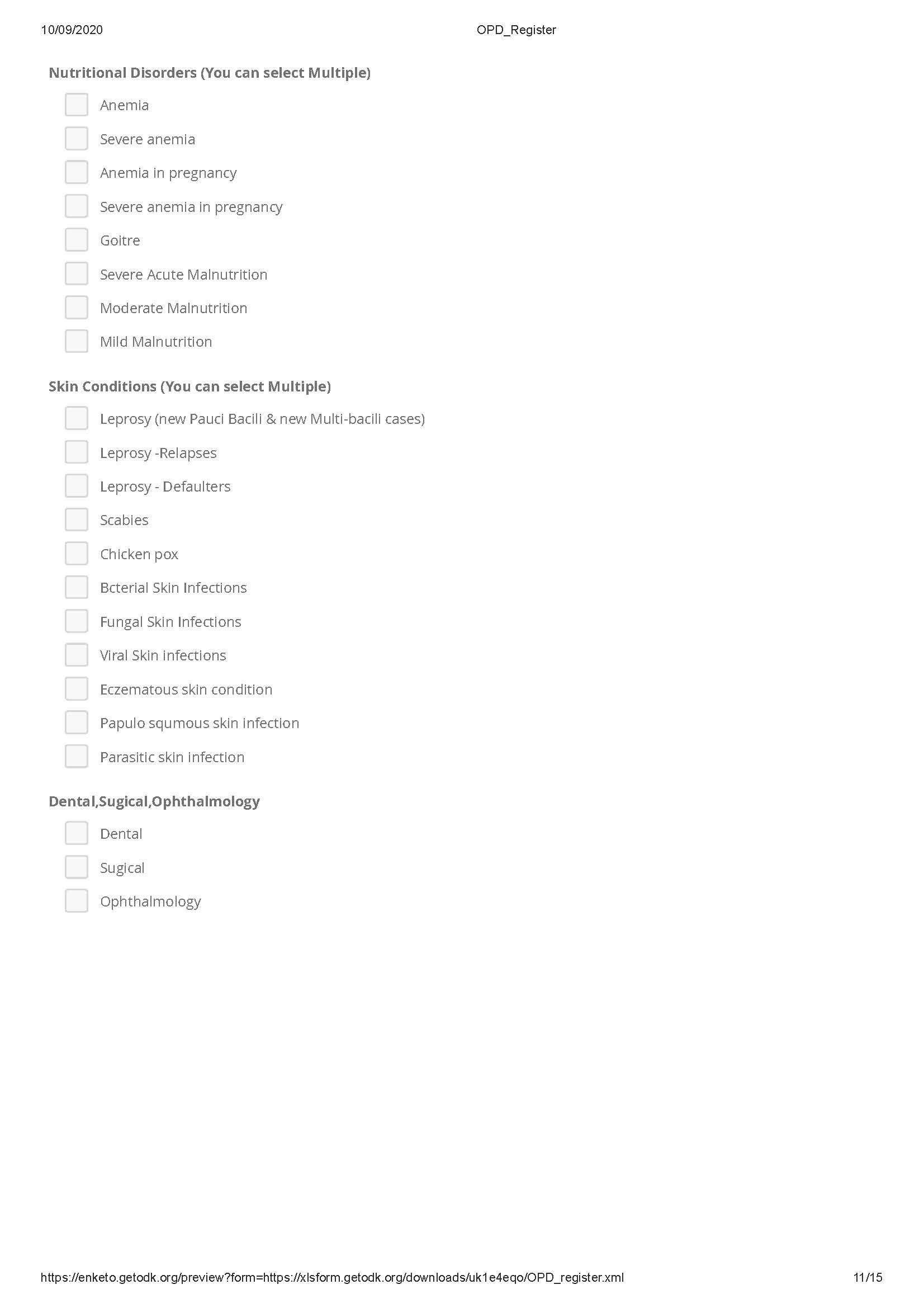

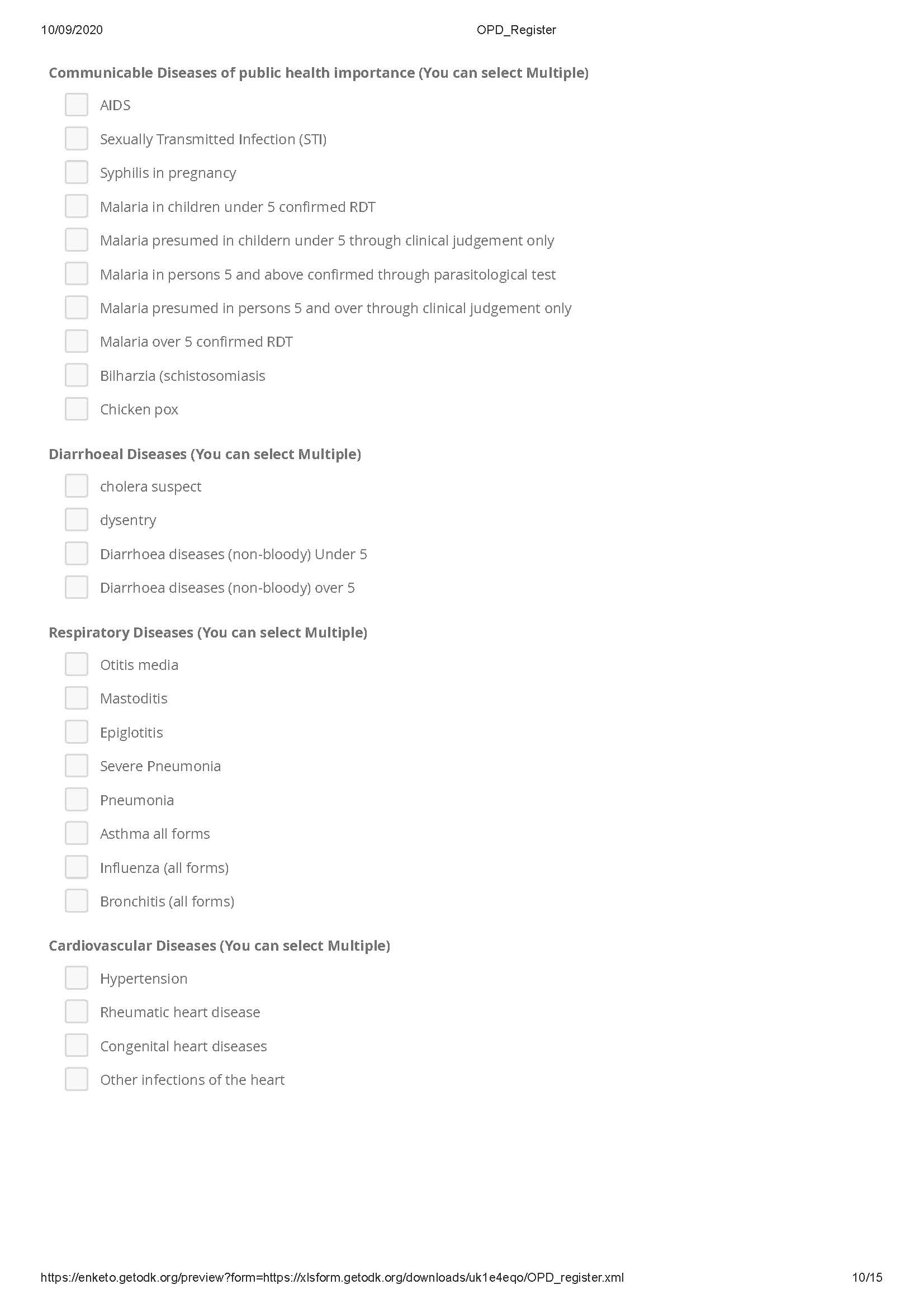

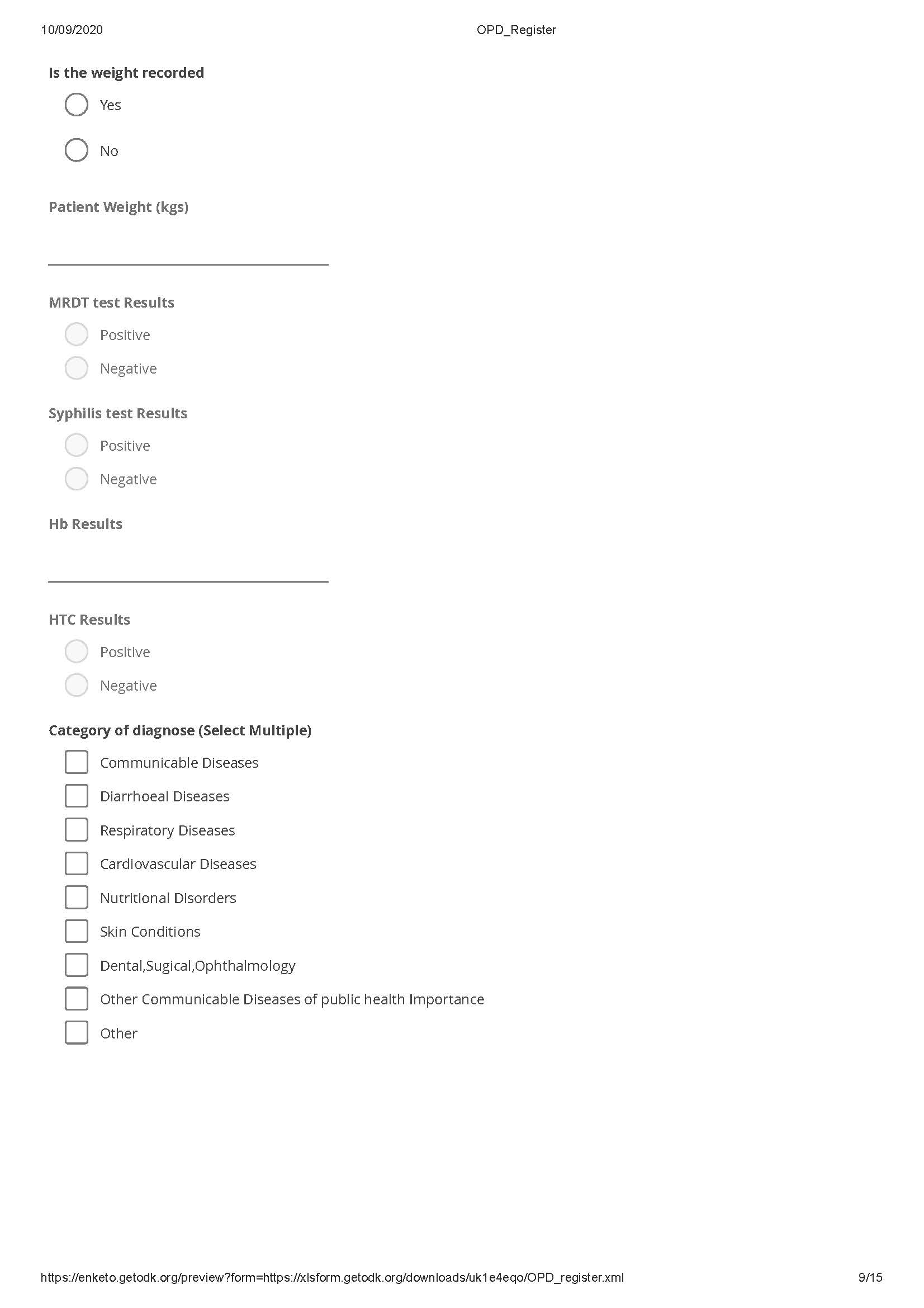

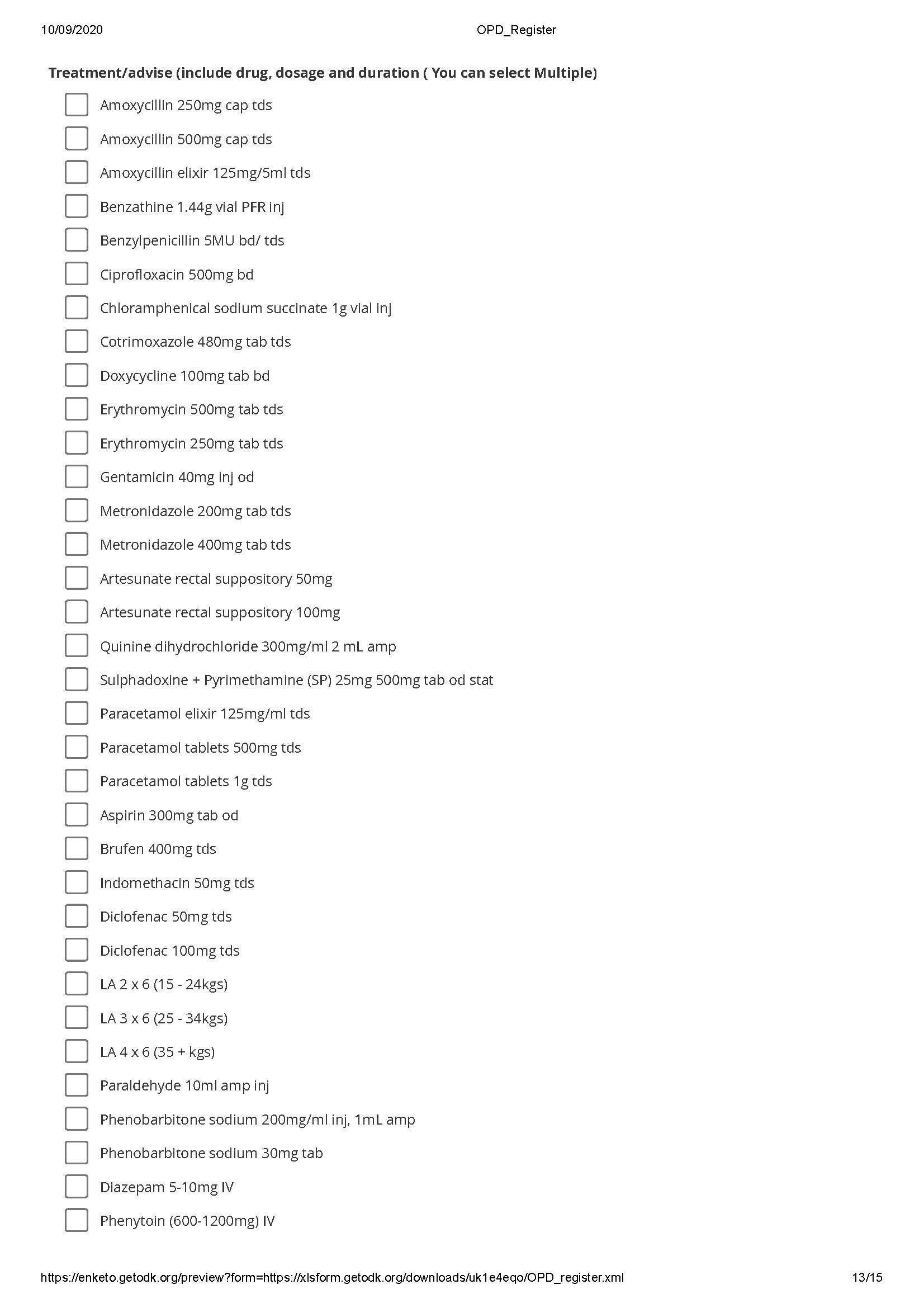


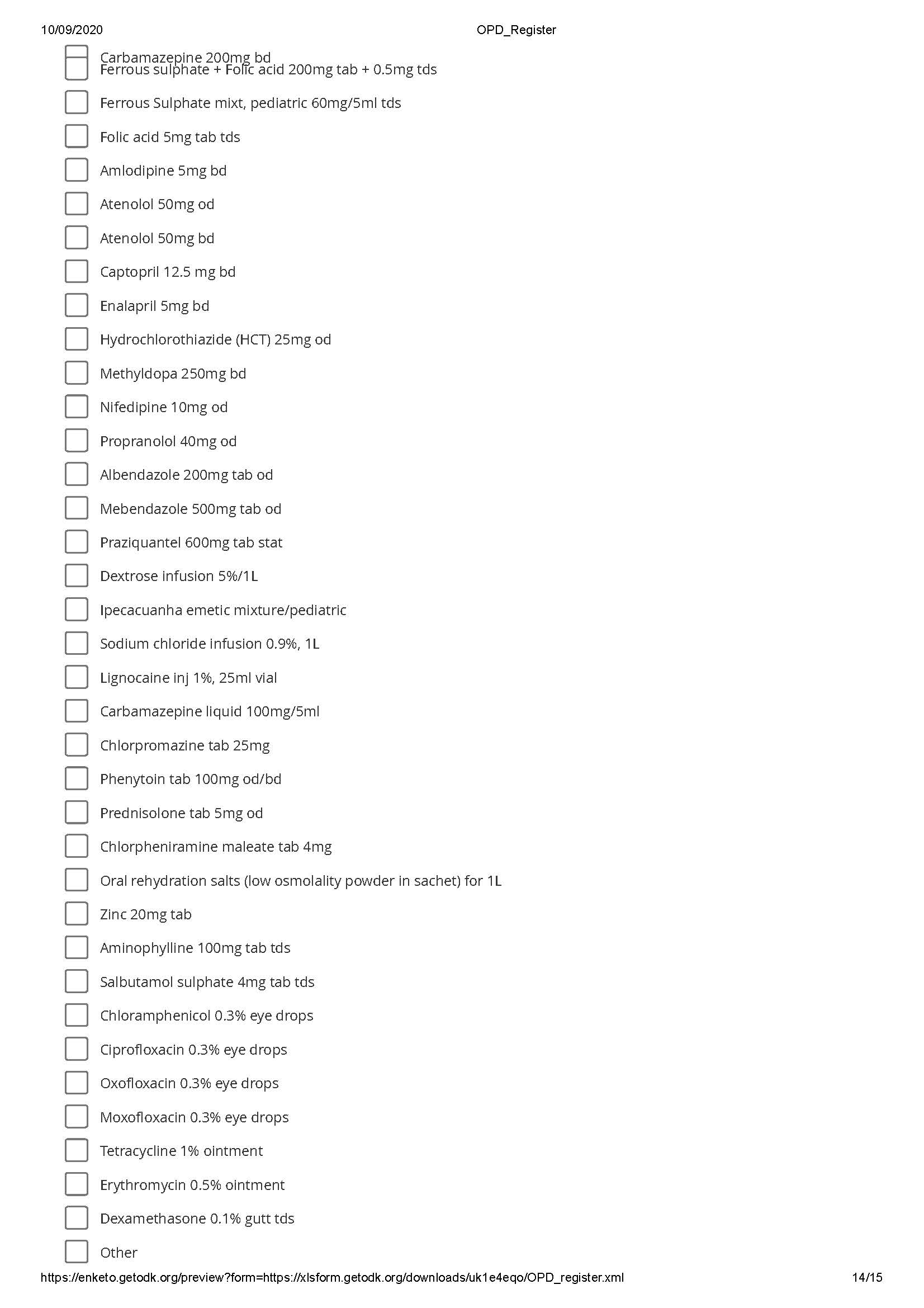

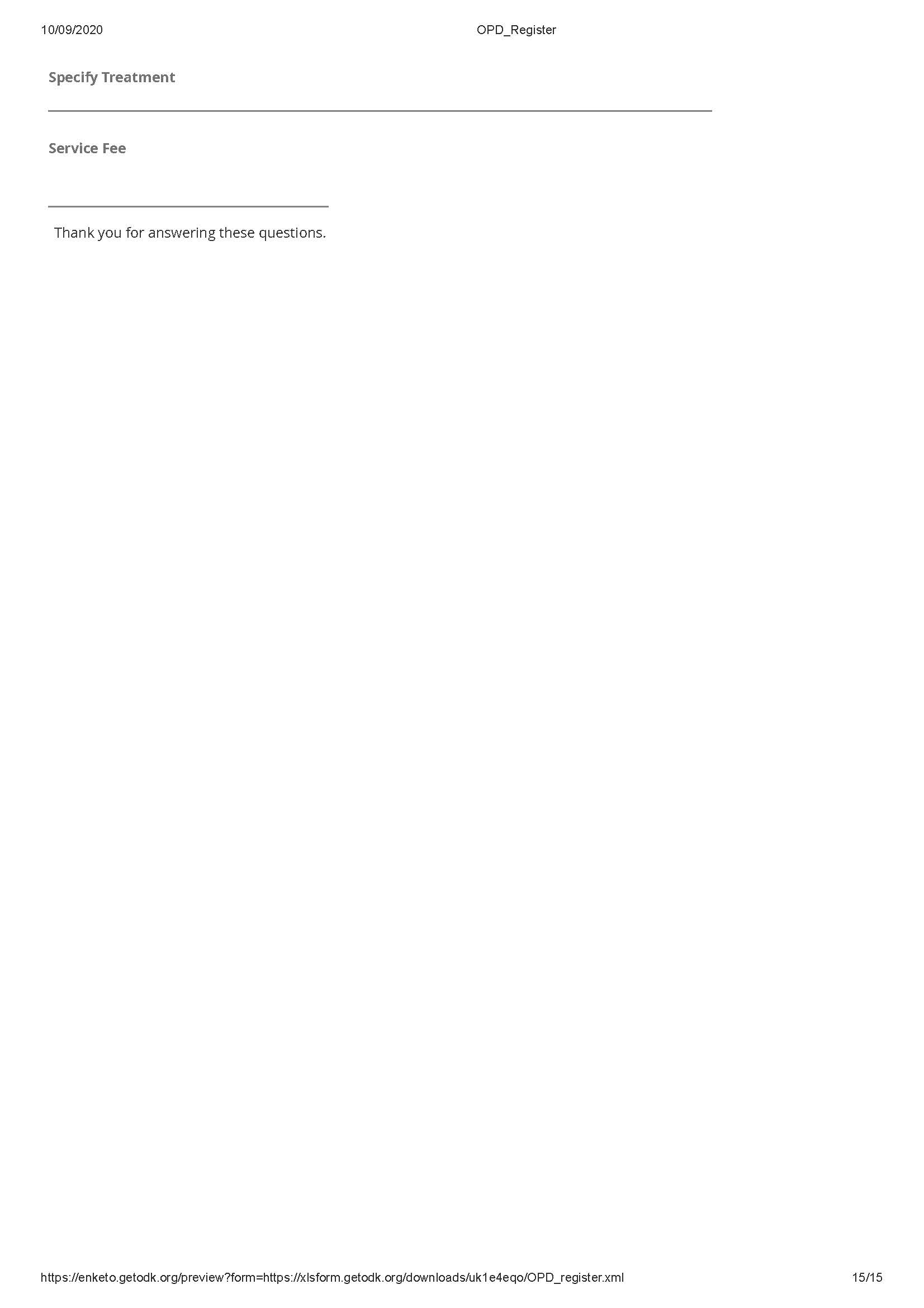


**Appendix 3:** Contents of the registers in its paper-based format which was later used and adopted for eCRF.

Image 1: Section of the OPD register


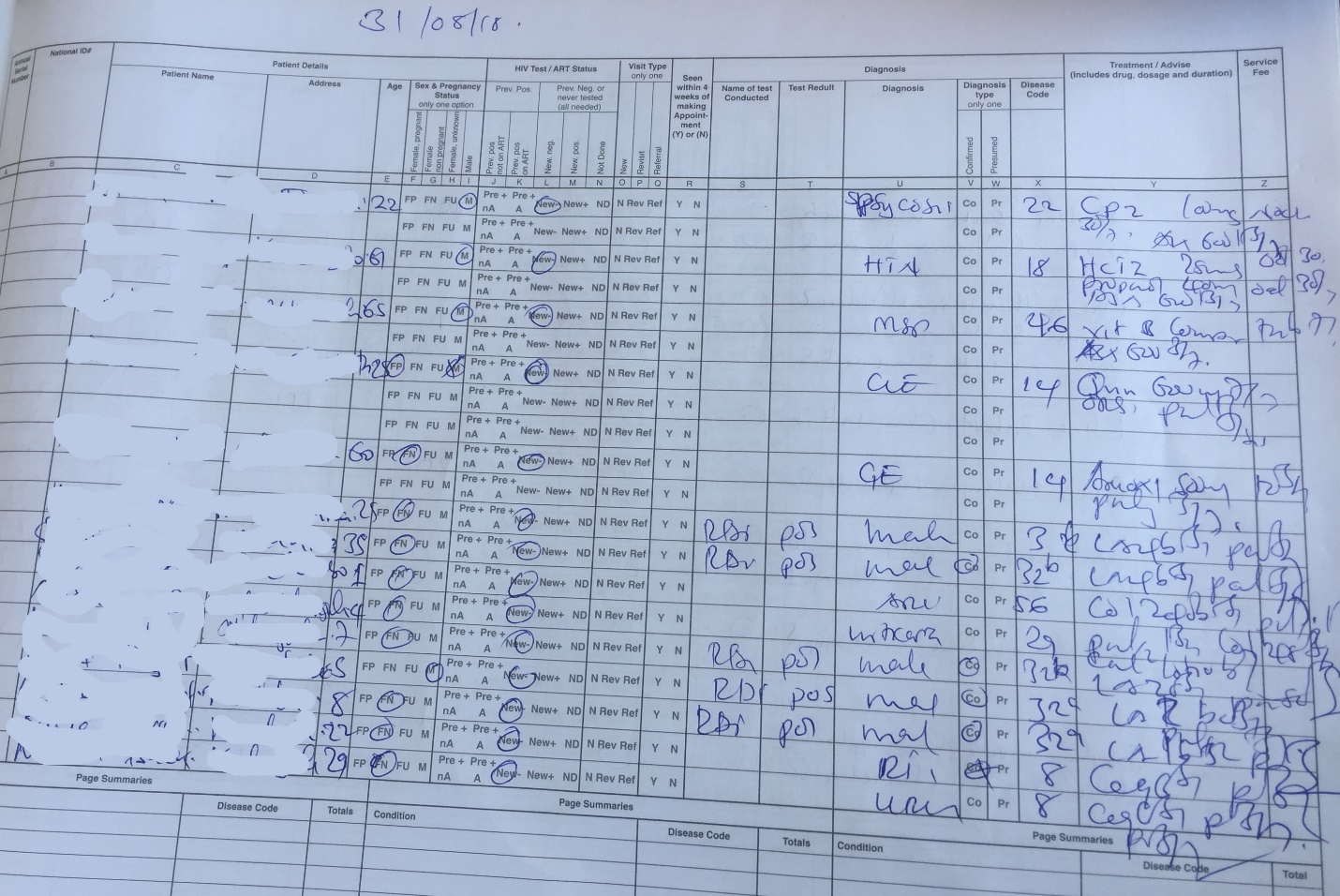


Image 2: Section of the OPD register

**
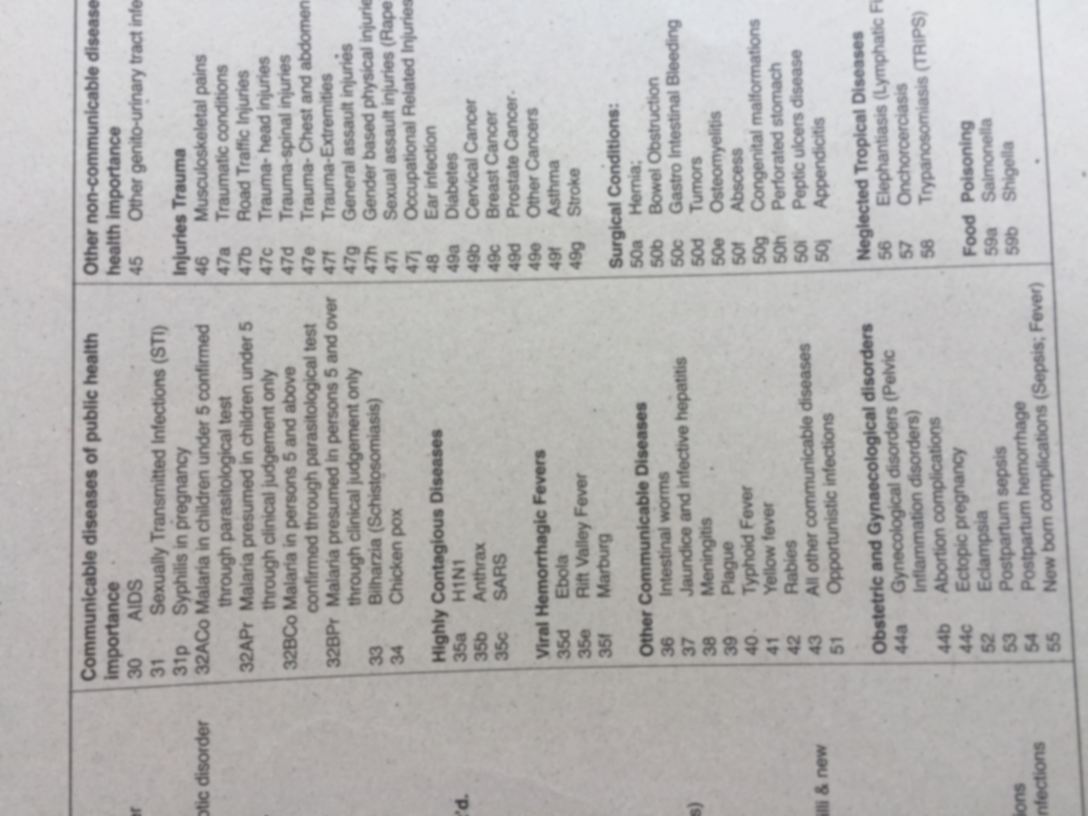
**

Image 3: Section of the malaria rapid diagnostic test (mRDT) register

**
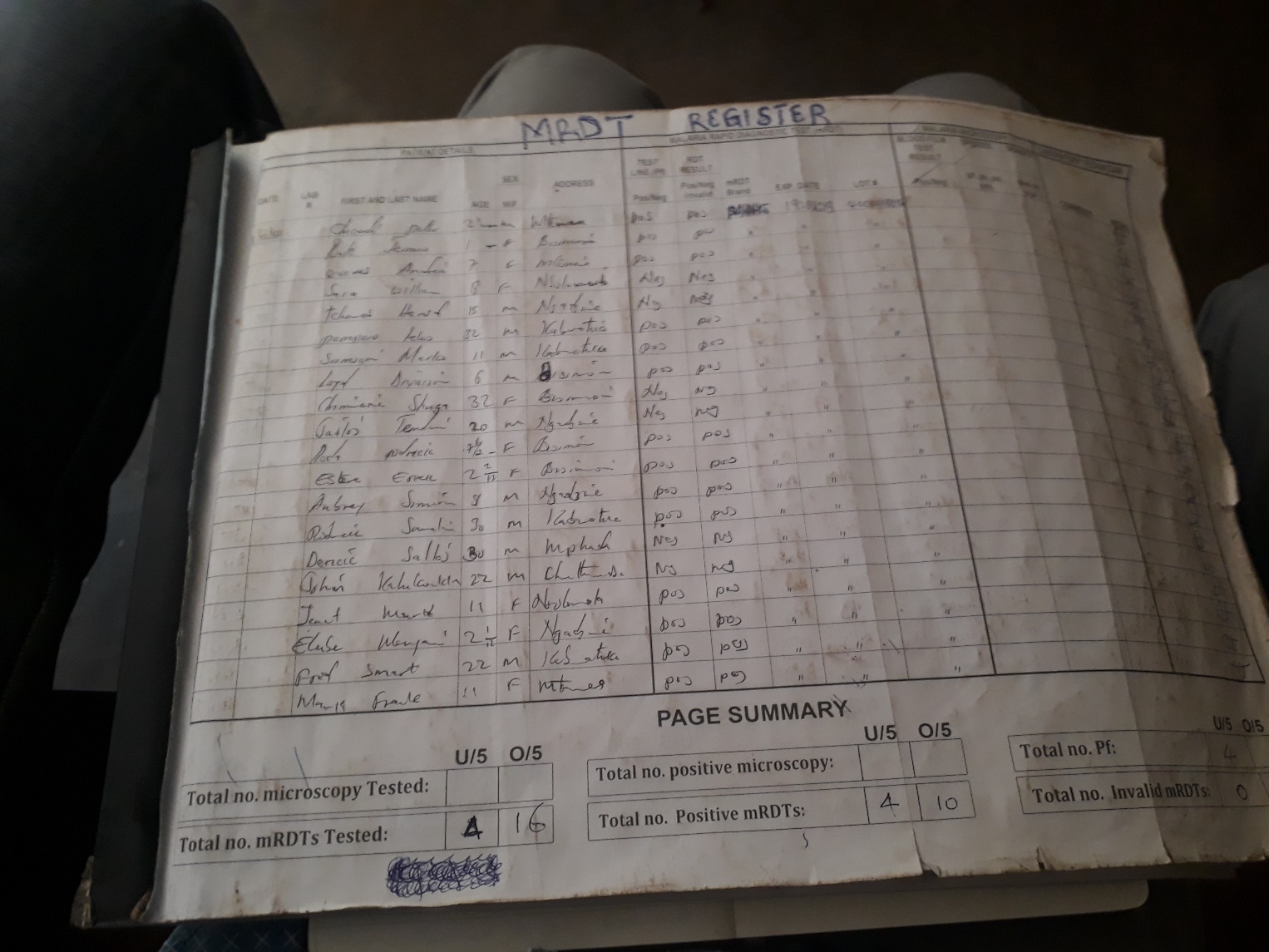
**

Image 4: Section of the OPD register

**
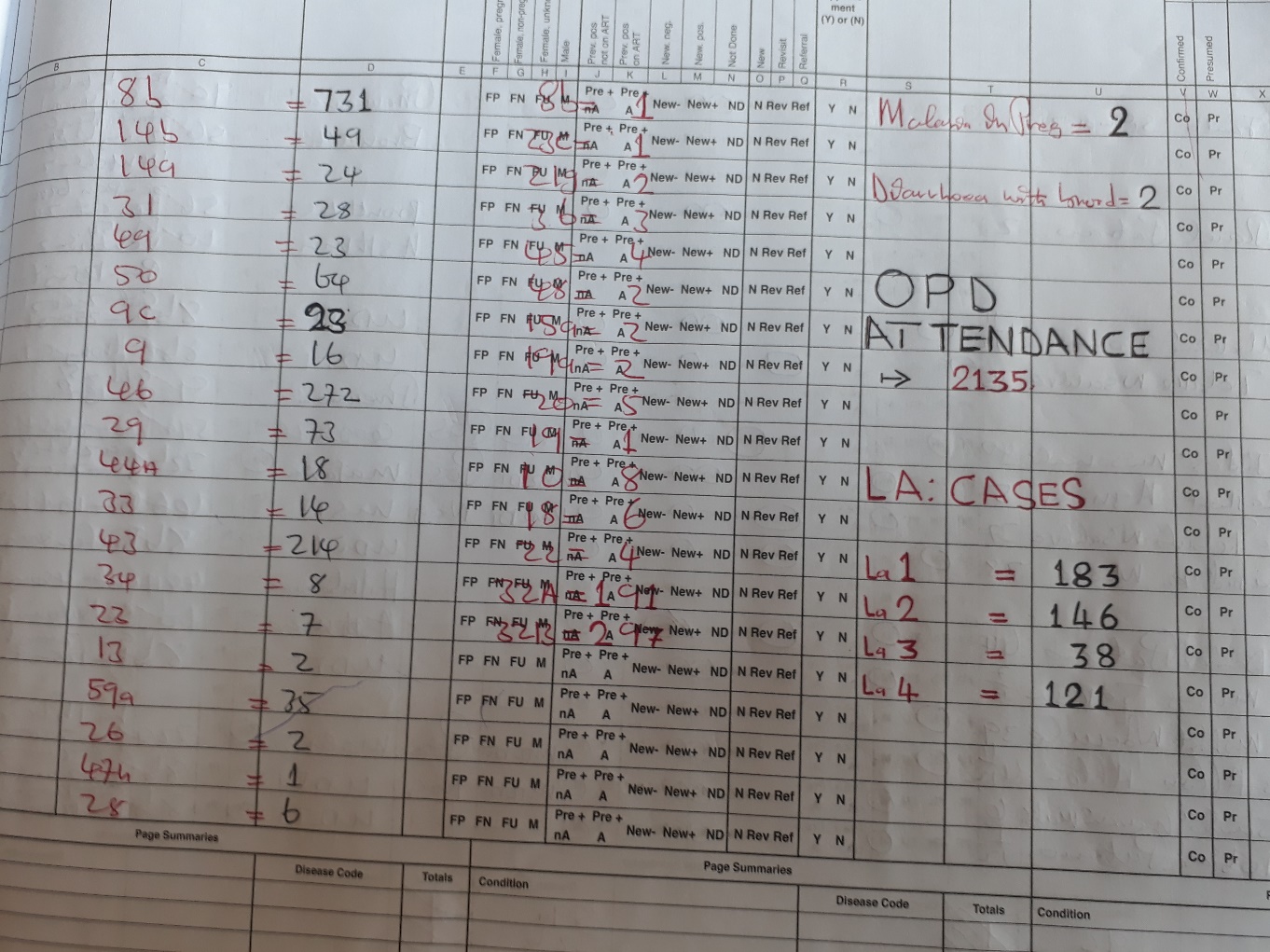
**

**Appendix 4:** Samsung tablets with ODK software used.


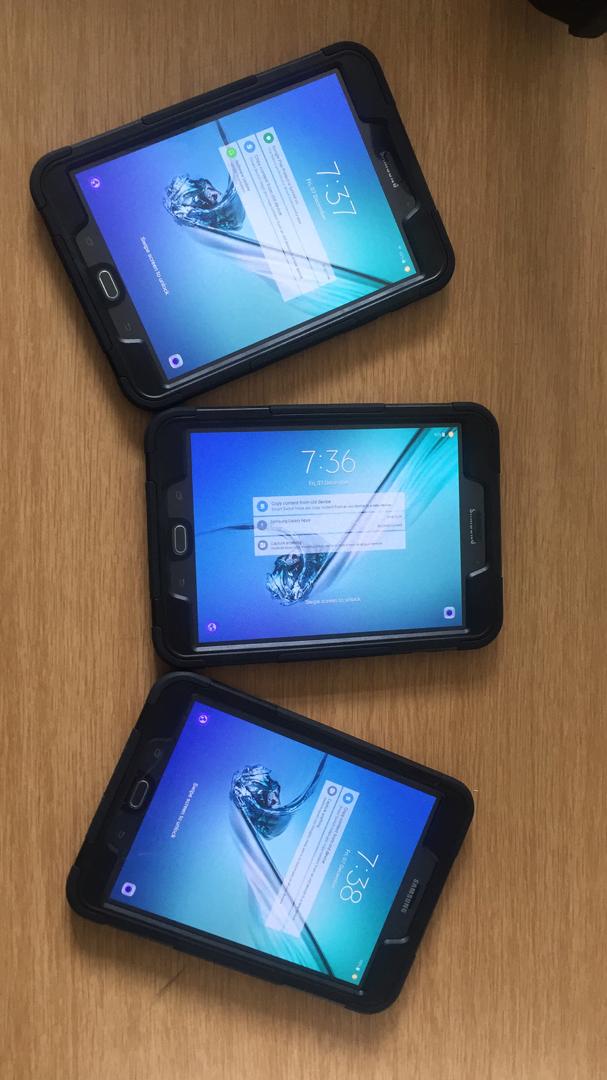


**Appendix 5:** Registers provided by the ministry of health, illegible print seen from registers, torn pages from registers

Image 1: Storage room containing various registers at Kakoma health center


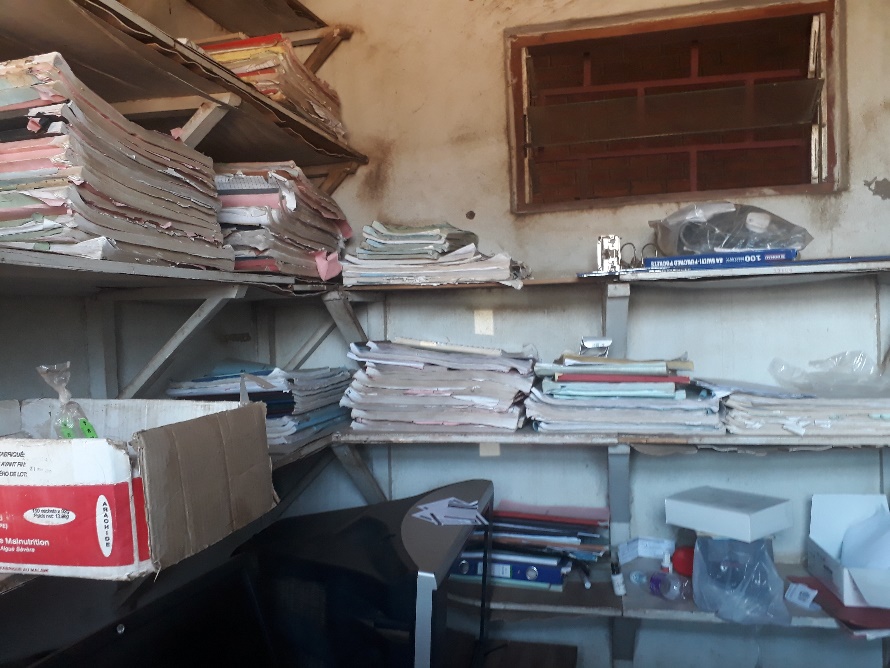


Image 2: Illegible print observed at the health facilities


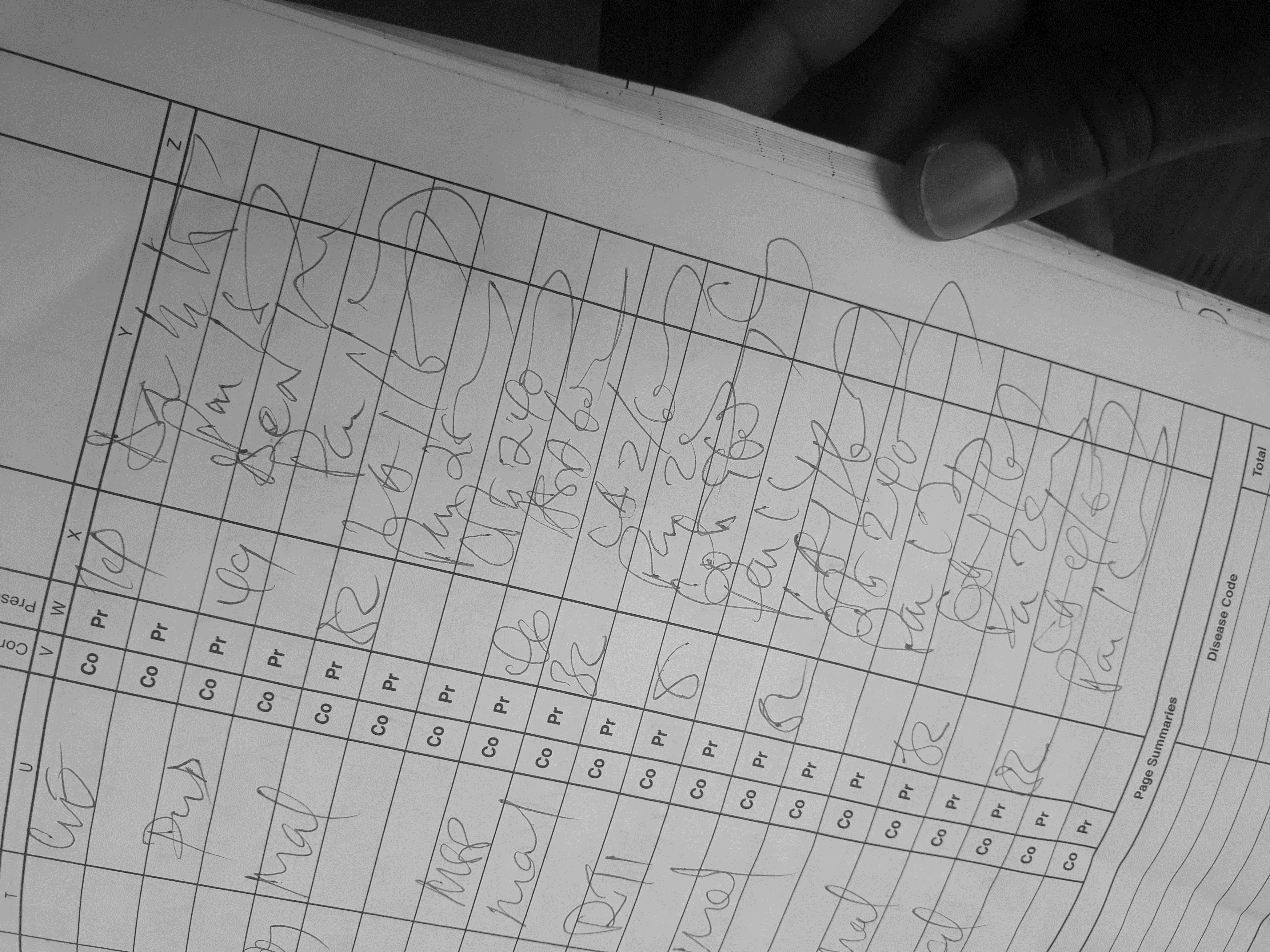


Image 3: Torn page of a register with illegible print


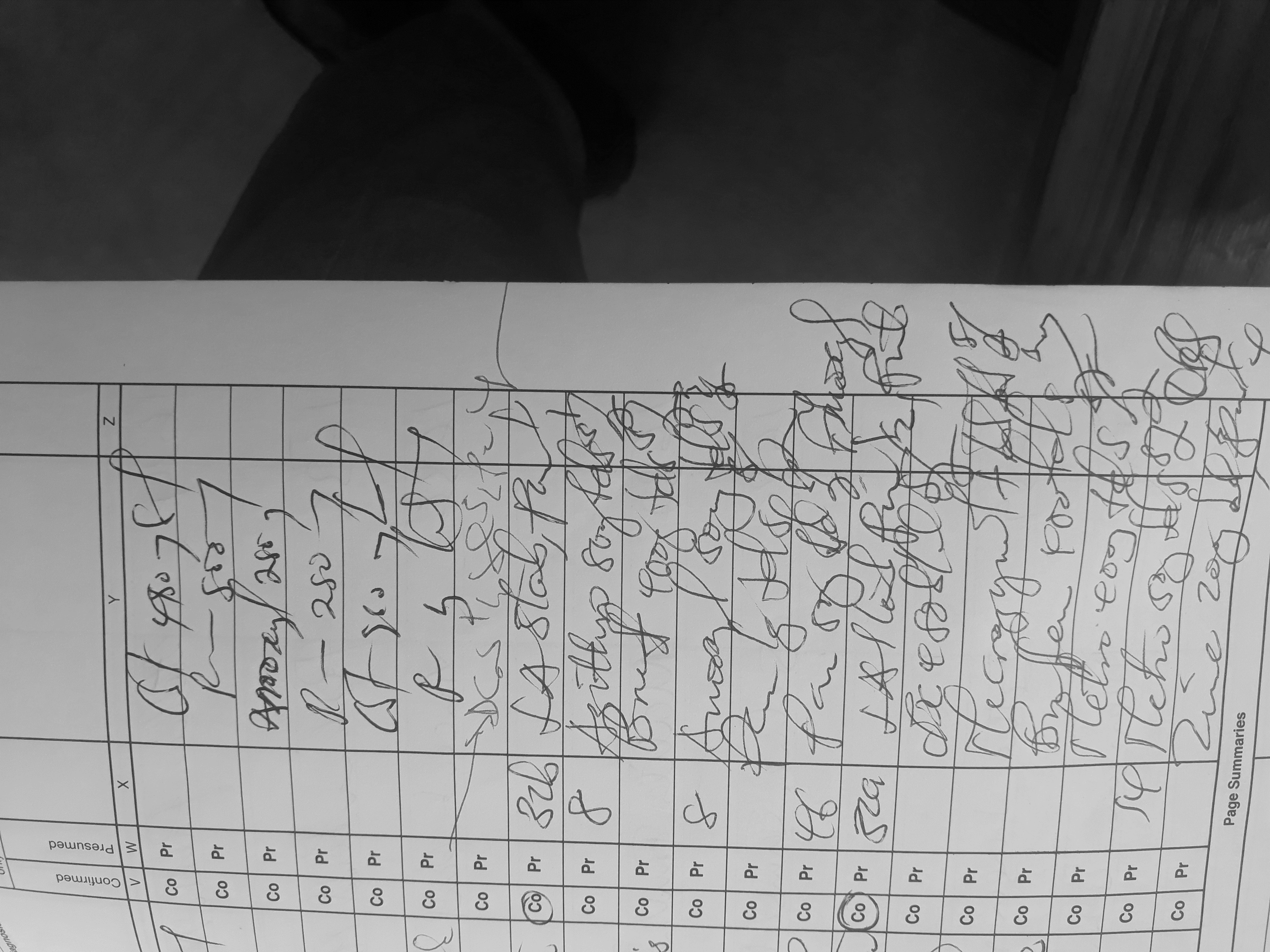

Supplement: Supplementary file 1 — Additional file 1: Appendix 1: Personnel and their roles in the project. Appendix 2: Electronic Case Report form (eCRF) adopted from registers. Appendix 3: Contents of the registers in its paper-based format which was later used and adopted for eCRF. Appendix 4: Samsung tablets with ODK software used. Appendix 5: Registers provided by the ministry of health, illegible print seen from registers, torn pages from registers. [file 12936_2021_3742_MOESM1_ESM.docx]
